# Prominent osteolysis in the maxilla: case report of an odontogenic fibroma mimicking a cyst

**DOI:** 10.1186/s12903-023-03008-9

**Published:** 2023-05-16

**Authors:** Clemens Raabe, Matthias Dettmer, Daniel Baumhoer, Valerie G. A. Suter

**Affiliations:** 1 Department of Oral Surgery and Stomatology, School of Dental Medicine, ZMK Bern, University of Bern, Bern, Switzerland; 2 Institute of Pathology, University of Bern, Bern, Switzerland; 3 Institute of Pathology, Klinikum Stuttgart, Katharinenhospital, Stuttgart, Germany; 4 Bone Tumor Reference Center at the Institute of Pathology, University Hospital Basel, University of Basel, Basel, Switzerland

**Keywords:** Odontogenic Tumors, Jaw Neoplasms, Osteolysis, Fibroma

## Abstract

**Background:**

Odontogenic fibroma (OF) is a rare benign odontogenic tumor of ectomesenchymal origin, mostly affecting the tooth-bearing portions of the jaws in middle-aged patients. Whilst small lesions tend to be clinically asymptomatic, varying unspecific clinical symptoms occur with an increase in size and may mimic odontogenic or other maxillofacial bone tumors, cysts, or fibro-osseous lesions of the jaws.

**Case presentation:**

A 31-year-old female patient presented with a hard, non-fluctuating protrusion in the vestibule of the upper right maxilla. It was visualized on cone beam computed tomography (CBCT) as space-occupying osteolysis with the displacement of the floor and facial wall of the maxillary sinus, mimicking a cyst-like lesion. The tissue was surgically removed and identified as an OF in the histopathological examination. One year after the surgery, restitution of regular sinus anatomy and physiological intraoral findings were observed.

**Conclusions:**

This case report emphasizes that rare entities, like the maxillary OF presented, often demonstrate nonspecific clinical and radiological findings. Nevertheless, clinicians need to consider rare entities as possible differential diagnoses and plan the treatment accordingly. Histopathological examination is essential to conclude the diagnosis. OF rarely recur after proper enucleation.

## Background

Odontogenic fibroma (OF) is a rare neoplasm of mature fibrous or fibromyxoid connective tissue occurring in the jawbones and accounting for < 1% of all odontogenic tumors [1–4]. The current WHO classification lists OF in the chapter on benign mesenchymal odontogenic tumors [4], including the even rarer amyloid, granular cell, ossifying, and hybrid subtypes that can show features of central giant cell granuloma [4]. According to the site of presentation, central and peripheral OF can be distinguished.

The OF usually represents a locally aggressive lesion originating from ectomesenchymal elements of the tooth-forming structures with unknown etiology and molecular pathogenesis [4–8]. The reported mean age for the diagnosis of OF is 34 years, although all age groups (range 3 – 80 years) may be affected [6]. Slight predilections were found for females (2.2:1 males) and the maxilla (53%, mandible 47%) [4, 8]. In the mandible, OF is predominant in the molar region (58%), followed by the premolar region (39%), and the ramus (26%). In the maxilla, OF is usually located in the premolar region (63%), followed by the incisor region (49%) and the molar region (19%) [6]. Lesion sizes of 3 – 130 mm (mean 28 mm) were reported in a recent systematic review of cases [7]. Usually, small lesions tend to be clinically asymptomatic and present as well-defined unilocular radiolucencies with corticated margins in the radiological examination [4, 7, 8]. With an increase in size, pain, expansion, or depression of the alveolar bone, and displacement or loosening of teeth are frequently observed symptoms [4, 7, 8]. More expansive lesions may present as multilocular radiolucencies with signs of cortical expansion in the radiological examination [4, 7, 8]. Furthermore, root resorption can occur [8]. More rarely, a mixed radiolucent-radiopaque appearance has also been described in the literature [8, 9].

As the clinical and radiologic features are non-specific and may mimic odontogenic or other maxillofacial bone tumors, cysts, or fibro-osseous lesions of the jaws, OF represents diagnostic challenges for the clinician. Histopathological examination is mandatory for proper diagnosis and demonstrates mature fibrous connective tissue with a proliferation of bland spindle cells. Varying amounts of inactive-looking odontogenic epithelium may be present or completely absent [9] Focal calcifications are commonly observed [4].

Generally, enucleation with preservation of surrounding structures is the treatment of choice for OF. Recurrences only rarely occur and are generally related to incomplete removal of the tumor [8, 9].

This case documents the rare occasion of an OF in the upper jawbone with expansion into the maxillary sinus, presenting clinical and radiographic aspects that resemble those of odontogenic cysts or other osteolytic lesions.

## Case presentation

A 31-year-old female patient felt a slowly growing swelling and mild pain in the right maxilla and consulted her private dentist, who performed a local incision and drainage. As symptoms persisted, the dentist referred the patient to the Department of Oral Surgery and Stomatology at the University of Bern. The patient was a smoker (10 cigarettes per day, cumulative dose of 10 pack years). Chronic bronchitis and penicillin intolerance were noticed in the medical history.

**Fig. 1:**
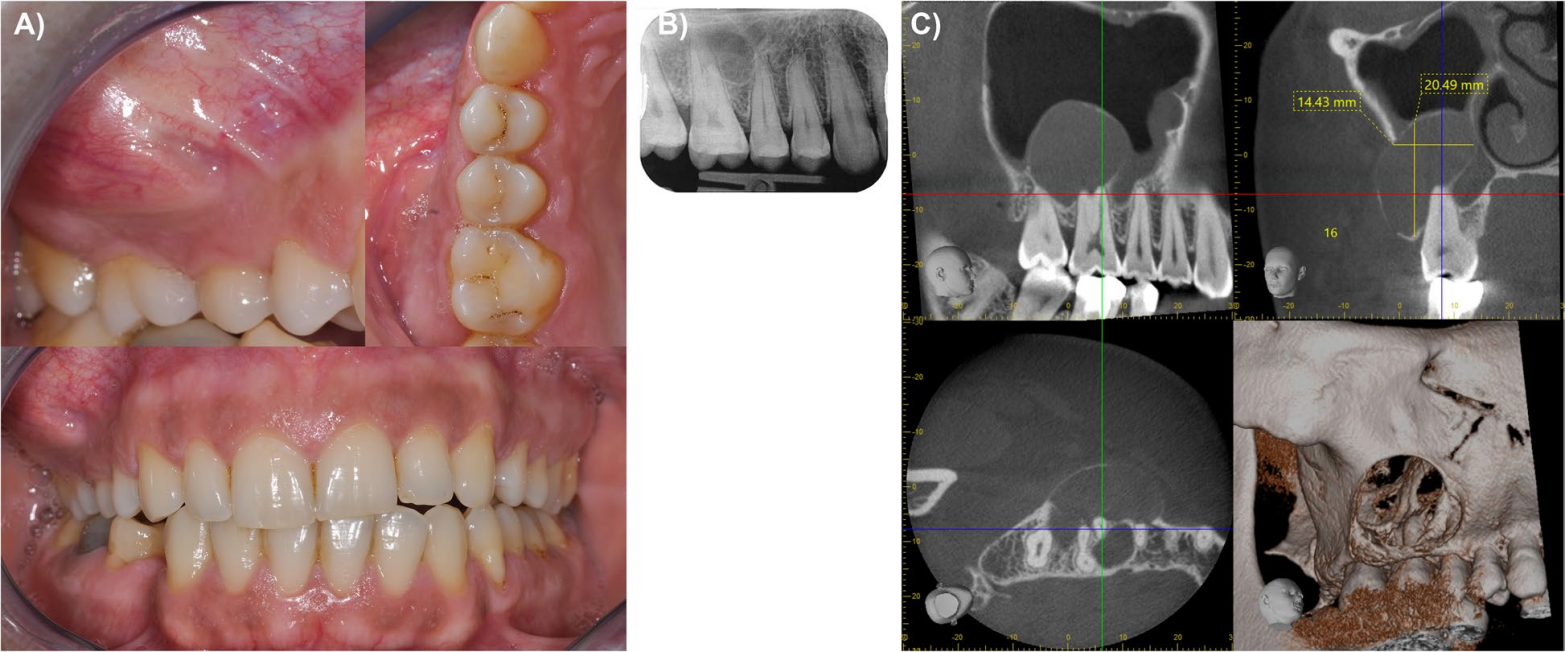
Initial clinical presentation including a hard, non-fluctuating and painless swelling in the vestibule of the right maxilla **A**. In the periapical radiograph, a well-demarcated radiolucency was visible in the apical region between the roots of the upper second premolar, first and second molar **B**. The preoperative CBCT showed space-occupying ovoid osteolysis in the right maxilla expanding into the maxillary sinus lumen and to the buccal vestibule **C**

The clinical head and neck examination showed a mild prominence of the right cheek and intact sensorimotor functions. No lymph nodes were palpable. Intraorally, a hard, non-fluctuating, and painless swelling in the vestibule of the right maxilla (FDI regions 15–17) was present (Fig. 1A). The dentition was unsuspicious, all teeth in the right maxilla showed sensitivity upon CO_2_ dry ice testing, physiological mobility, and regular probing depths.

In the periapical radiograph, a well-demarcated radiolucency was visible in the apical region between the roots of the upper second premolar, first and second molar (Fig. 1B). Subsequently, a cone beam computed tomography (Accuitomo 170; Morita Corp., Kyoto, Japan, field of view 6 × 6 cm, 90 kV, 5 mA, 180°) was obtained which showed a space-occupying ovoid osteolysis in the right maxilla (30 × 20x12 mm). The osteolysis expanded into the maxillary sinus as well as the the buccal vestibule and was demarcated by a thin radiopaque line with small interruptions. Direct contact with the roots of the second premolar, first and second molar, but no visible signs of resorptions or displacement of the roots were noticed (Fig. 1C).

Clinically and radiologically an odontogenic keratocyst was most likely suspected. Although predominantly affecting the lower jaw, 23% of odontogenic keratocysts occur in the maxilla with a predilection to the molar region [10]. Although a lateral periodontal cyst, a central giant cell granuloma, and odontogenic tumors were among the differential diagnoses considered, they were deemed less likely in this case.

The planned surgical treatment consisted of the enucleation of the entire lesion and its histopathologic examination. Anticipating intraoperative findings potentially indicating malignancy, such as irregular tumorous tissue presentation, destruction, or invasion of adjacent structures, an incisional biopsy was considered the preferred method. As devitalization of the first and second molar during surgery was highly probable, root canal treatments were performed before surgery.

**Fig. 2:**
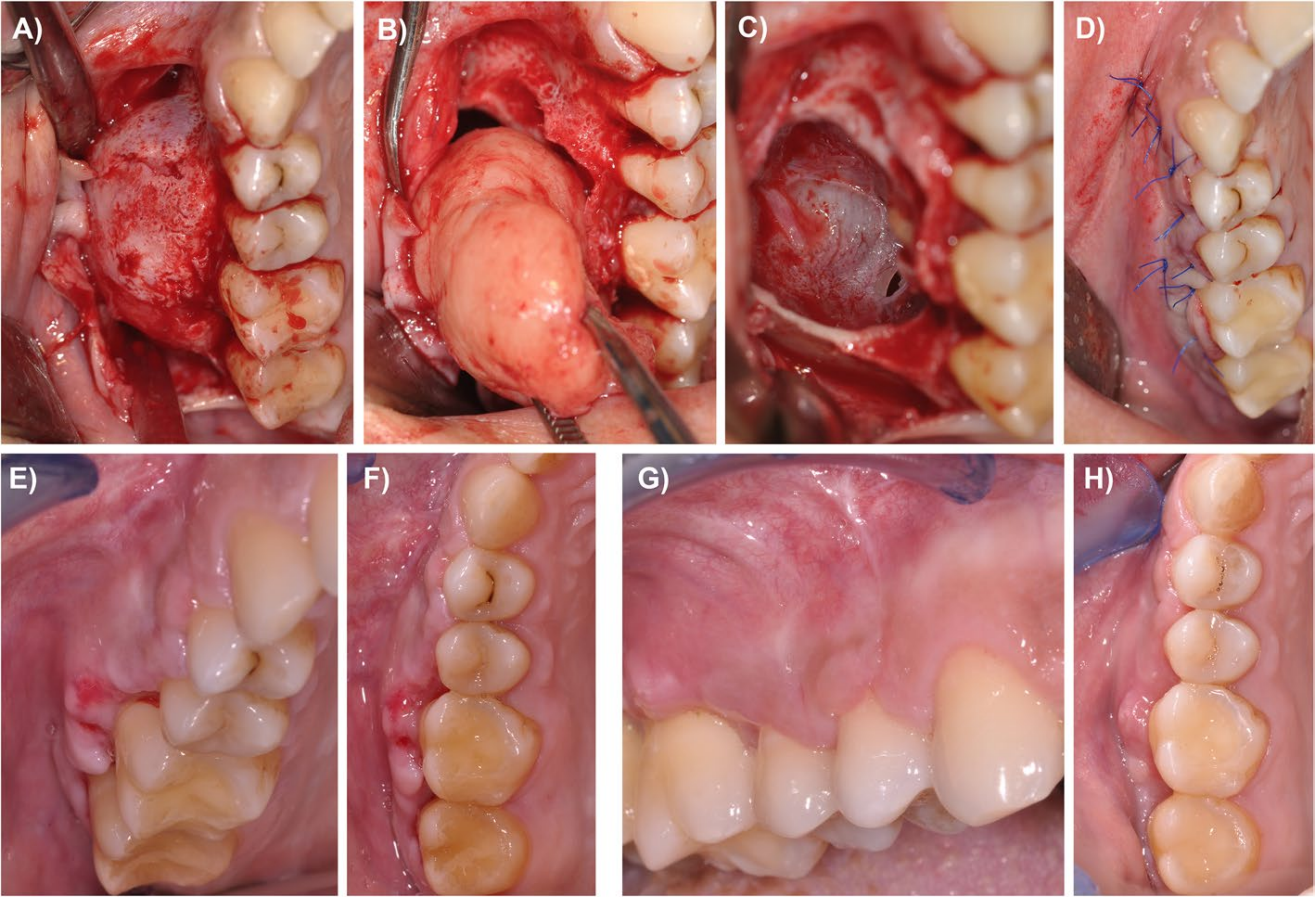
Preparation of a mucoperiosteal flap and presentation of the lesion **A**. Blunt dissection and enucleation of the tumor **B**. A small rupture of the maxillary sinus mucosa presented medially, which was left in place **C**. Primary wound closure **D**. Lateral and occlusal view one week (**E**, **F**) and two weeks postoperatively (**G**, **H**)

After the onset of the local anesthesia, the surgery was initiated by a buccal sulcular incision and the preparation of a mucoperiosteal flap (Fig. 2A). Subsequently, the lesion was outlined by osteotomy. Upon presentation of a well-defined capsule and no invasion of adjacent structures, the surgeons agreed to perfom complete enucleation. Subsequently, the tumor was dissected bluntly from the surrounding tissues (Fig. 2B). The yellow tissue showed a surprisingly rubbery-solid texture upon palpation. It was removed in toto and fixed in formalin 4% for subsequent histopathological examination. Due to the intrasurgical presentation and structure, a keratocyst was ruled out and a solid odontogenic tumor was considered the most likely diagnosis. After the resection, the inspection of the surgery situs revealed a small rupture of the maxillary sinus mucosa medially, which was left in place (Fig. 2C), and primary wound closure was performed (Fig. 2D). The patient was prescribed postoperative antibiotics, analgesics and a decongestant nasal spray. She was instructed on the usual postsurgical measures and not to blow her nose.

One week after the surgery, wound healing was slightly retarded with redness of the marginal gingiva (Fig. 2E, F) whereas two weeks postoperatively, the patient showed regular wound healing (Fig. 2G, H).

**Fig. 3:**
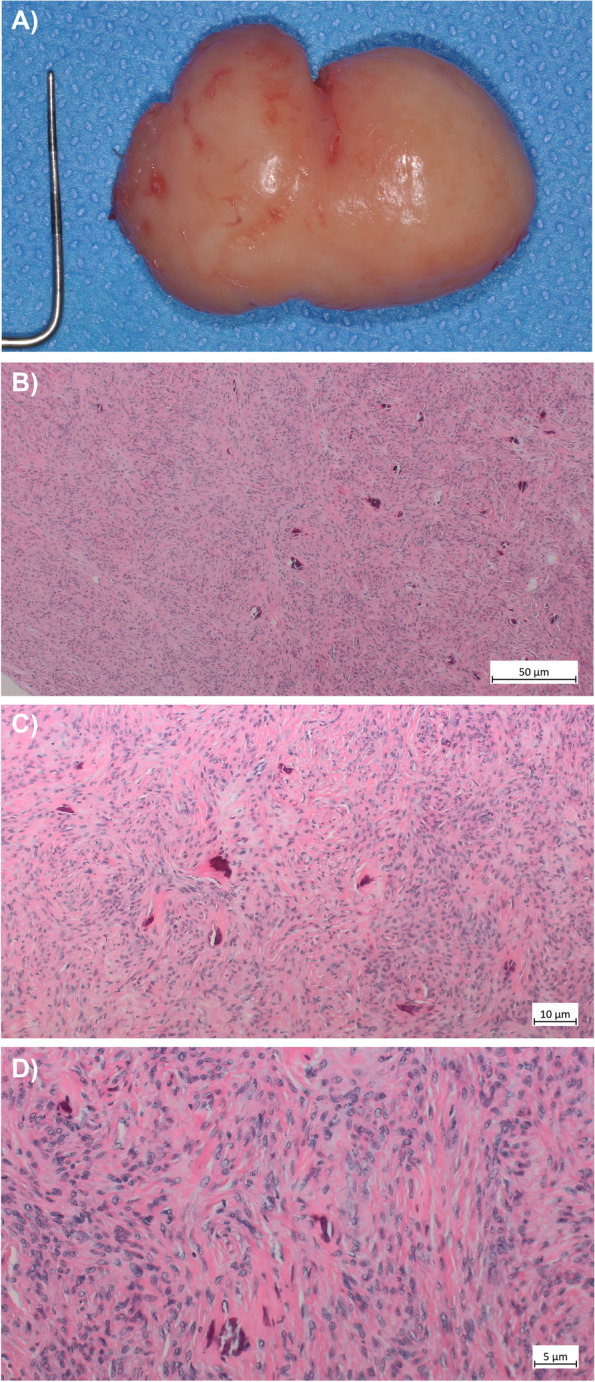
Macroscopically, the tumor presented as a beige, firm and well-demarcated mass **A**. The histology shows a cellular proliferation of bland, clumsy, and monomorphic spindle cells in a collagenous background with focal dystopic calcifications, and a partially storiform growth pattern. Increased mitotic activity, necrosis and atypia were not apparent, also no epithelial component was noted. 4x (**B**), 10x (**C**) and 20 × magnification, all hematoxylin and eosin (H&E) stainings (**D**)

Due to the rarity of the finding, the biopsy was evaluated by pathologists at both Institutes of Pathology of the University of Bern and the Bone Tumor and DOESAK (German-Swiss-Austrian Working Group of Maxillofacial Tumors) reference registry in Basel. The specimen showed a proliferation of monomorphic spindle cells with focal dystopic calcifications and a background rich in collagen fibers. An epithelial component was ruled out by an immunohistochemical stain against pan-cytokeratin (AE1/3). Considering the clinical-radiological context, the diagnosis of an odontogenic fibroma was made (Fig. 3A-D).

**Fig. 4:**
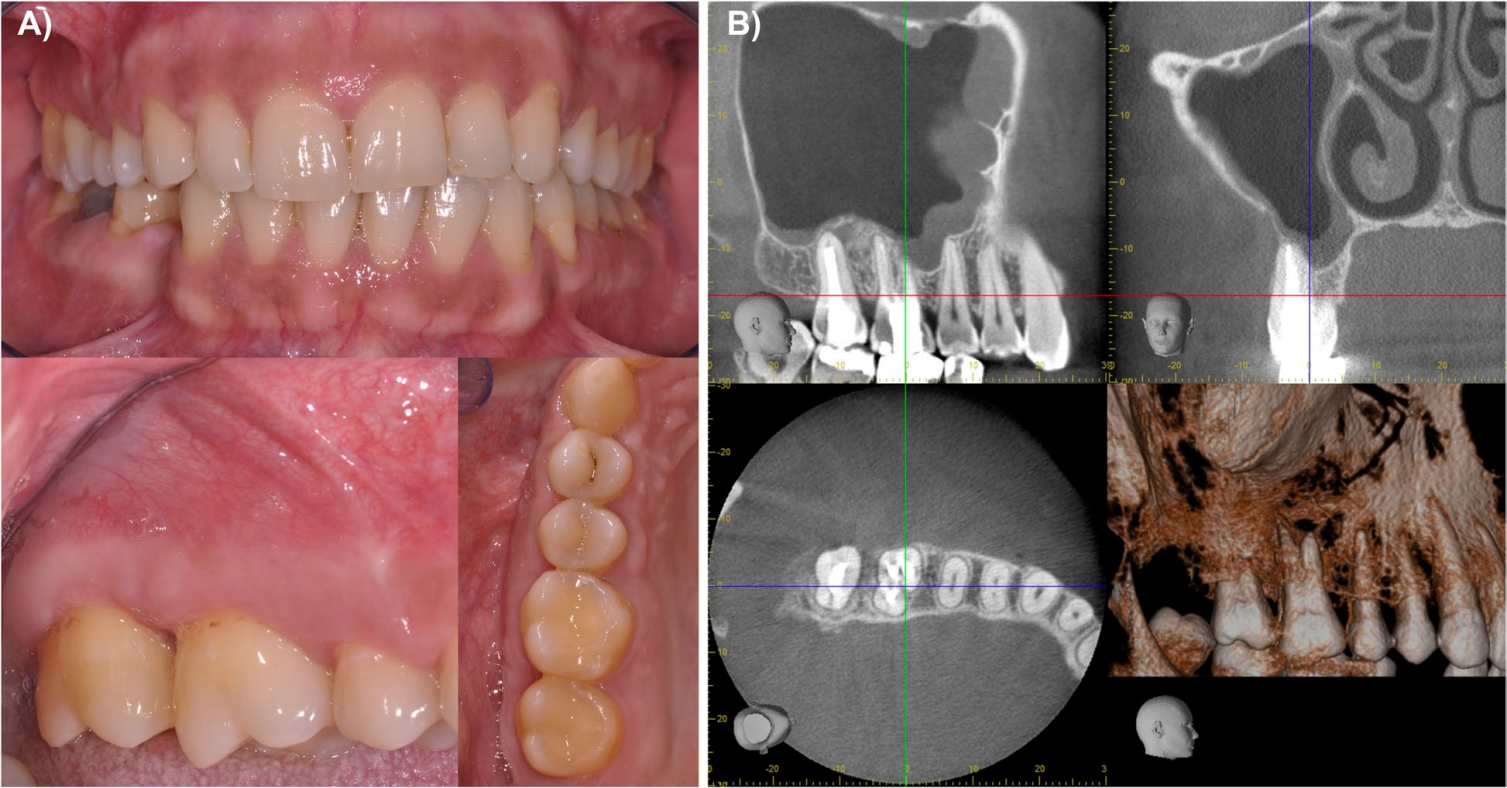
Normal findings in the clinical presentation after six months **A**. The follow-up CBCT showed the restitution of the regular sinus anatomy with a small residual defect in the facial maxillary sinus wall **B**

Six months after the surgery the patient was symptom-free. The intraoral clinical examination presented normal findings (Fig. 4A). The CBCT (6 × 6 cm, 90 kV, 5 mA, 180°) showed the restitution of the regular sinus anatomy with a small residual defect in the facial maxillary sinus wall and circumferential flat thickening of the sinus membrane (Fig. 4B). One year postoperatively, a clinical examination showed physiological and unsuspicious intraoral findings. Due to the patient’s pregnancy, no radiological examination was performed. Further annual clinical and radiological control examinations are scheduled to exclude a possible recurrence.

## Discussion and conclusions

This case report documents the treatment of a central odontogenic fibroma in the right maxillary sinus, which presented primarily as a non-specific expansive osteolysis of the maxilla mimicking a cyst.

Historically, odontogenic fibroma had already been included in the first WHO classification in 1971 with changing definitions and classifications over the years. In the current WHO classification, different histopathological subtypes were added [4]. Additionally, and in contrast to the prior editions, the odontogenic epithelium is no longer required to make the diagnosis. A recent systematic review of the literature identified reports of 135 central OFs until the year 2021 [6]. Given its rarity, clinicians may not be adequately familiarized with OF and therefore fail to consider it as differential diagnosis.

OF may be associated with a variety of symptoms. The presented patient noticed a prominence of the right cheek, mild pain, and a slowly progressing swelling of the right maxilla. According to Bennabi et al. [11], extraoral symptoms are absent in 75% of OF, whilst facial asymmetries (24%), lymphadenopathies (1.5%), or paresthesia (0.7%) occurred infrequently. Intraorally, OF commonly presented slowly progressing swellings (58%), and rarely palatal depressions (2.2%) were observed. Dental symptoms like tooth mobility, delayed eruption, or pulp necrosis were other signs found in conjunction with OF in 18% of cases [6–8].

In the radiological examination, the current case presented as unilocular space-occupying osteolysis in the right maxilla with displacement and local small perforations of the floor and facial wall of the maxillary sinus. The majority of OF present as unilocular (54.1%), or multilocular (23.7%) radiolucencies. However, mixed radiolucent-radiopaque lesions (11%) have also been described. Additional radiological findings are corticated margins (37%), tooth displacement (44%), root resorptions (24%) or cortical bone perforations (16%) [6].

Although the appearance of OF is locally aggressive, the growth rate seems to be limited in most cases [8] and malignant transformations of OF have not been described so far [6, 12]. The non-specific radiological and clinical features observed in the present case suggest a range of differential diagnoses, including other odontogenic tumors such as epithelial (e.g. ameloblastoma), mixed epithelial-mesenchymal (e.g. ameloblastic fibroma), or mesenchymal variants (e.g. myxoma, cemento-ossifying fibroma). Particularly unilocular odontogenic myxoma, also originating from ectomesenchyme and accounting for 3–6% of all odontogenic tumors, might show similarities [11, 13]. Additionally, cysts (e.g. odontogenic keratocyst, lateral periodontal cyst), or osteolytic lesions such as giant-cell lesions should also be included as potential differential diagnoses. Therefore, a histopathological examination is crucial for patient management and the correct diagnosis in the context of radiological imaging and clinical presentation. Immunohistochemistry is usually not required, but stains against cytokeratins can be used to detect single epithelial cells that might be difficult to identify in hematoxylin and eosin (H&E) stains.

Treatment of OF usually consists of full enucleation with preservation of the adjacent teeth and periodontium [5]. In some cases, tooth extraction or apicoectomy was indicated to ensure access to the tumor [6]. Recurrences of OF were observed in approximately 6—10% of cases [7, 8, 14] and discussed to be associated with incomplete surgical removal [14, 15]. Increased risk for recurrence was found for OF located in the maxilla compared to the mandible, lesions perforating the cortical bone compared to non-perforation and multilocular lesions compared to unilocular lesions [7]. While resective surgery (up to interruptive mandibulectomy) has been performed in some cases and might reduce the risk of recurrence [7], complex defect reconstruction is necessary afterward [6]. Alternatively, an initial incisional biopsy and histopathological examination have to be considered in case of doub to ensure the diagnosis before further treatment planning and execution [16]. In the presented case, the conservative enucleation of the pathology did facilitate the restitution of the regular sinus anatomy six months postoperatively and no recurrence was noticed. However, a small defect was visible in the facial wall of the maxillary sinus, which could be a sign of both ongoing regeneration, or residua of the intervention.

Ideally, a follow-up X-ray would have been taken one year after the surgical enucleation, but it was not indicated due to the patient`s pregnancy. Subsequently, the patient relocated, which precluded additional visits. Therefore, the major limitation of this case report is the lack of long-term follow-up to identify a possible recurrence of the pathology.

In conclusion, clinicians must be aware that expansive osteolysis in the jawbone are compatible with several differential diagnoses including rare entities. Odontogenic fibromas are rare benign odontogenic tumors presenting with non-specific clinical symptoms and often associated with expansive osteolysis that mimics cyst-like lesions, odontogenic tumors, or other osseous lesions in the alveolar bone.

## Data Availability

The datasets used and/or analysed during the current study are available from the corresponding author on reasonable request.

## Abbreviations

OF: Odontogenic fibroma
WHO: World Health Organization

## References

[1] Brannon RB (2004). Central odontogenic fibroma, myxoma (odontogenic myxoma, fibromyxoma), and central odontogenic granular cell tumor. Oral Maxillofac Surg Clin North Am.

[2] Sriram G, Shetty RP (2008). Odontogenic tumors: a study of 250 cases in an Indian teaching hospital. Oral Surg Oral Med Oral Pathol Oral Radiol Endod.

[3] Mosqueda-Taylor A, Martínez-Mata G, Carlos-Bregni R, Vargas PA, Toral-Rizo V, Cano-Valdéz AM (2011). Central odontogenic fibroma: New findings and report of a multicentric collaborative study. Oral Surg Oral Med Oral Pathol Oral Radiol Endod.

[4] WHO Classification of Tumours Editorial Board. Head and neck tumours [Internet; beta version ahead of print]. Lyon (France): International Agency for Research on Cancer; 2022. (WHO classification of tumours series, 5th ed.; vol. 9). Cited 2022 12 09

[5] Pontes FSC, Mosqueda-Taylor A, de Souza LL, de Paula LP, Batista LAL, Rodrigues-Fernandes CI (2022). Hybrid odontogenic lesions: A systematic review of 203 cases reported in the literature. J Oral Pathol Med.

[6] Bennabi S, Lesclous P, Cloitre A (2021). Central Odontogenic Fibroma: Characteristics and management. J Oral Med Oral Surg.

[7] Correa Pontes FS, Lacerda de Souza L, Paula de Paula L, de Melo Galvão Neto E, Silva Gonçalves PF, Rebelo Pontes HA (2018). Central odontogenic fibroma: An updated systematic review of cases reported in the literature with emphasis on recurrence influencing factors. J Craniomaxillofac Surg.

[8] Roza ALOC, Sousa EM, Leite AA, Amaral-Silva GK, de Lima Morais TM, Wagner VP (2021). Central odontogenic fibroma: an international multicentric study of 62 cases. Oral Surg Oral Med Oral Pathol Oral Radiol.

[9] Van Heerden WFP, Kusama K, Neville BW (2017). Odontogenic fibroma. WHO classification of head and neck tumours.

[10] Boffano P, Cavarra F, Agnone AM, Brucoli M, Ruslin M, Forouzanfar T (2022). The epidemiology and management of odontogenic keratocysts (OKCs): A European multicenter study. J Craniomaxillofac Surg.

[11] Dotta JH, Miotto LN, Spin-Neto R, Ferrisse TM (2020). Odontogenic Myxoma: Systematic review and bias analysis. Eur J Clin Invest.

[12] Zhou CX, Li TJ (2018). A clinicopathologic study on central odontogenic fibroma: with special reference to amyloid variant. Oral Surg Oral Med Oral Pathol Oral Radiol.

[13] Chrcanovic BR, Gomez RS (2019). Odontogenic myxoma: An updated analysis of 1,692 cases reported in the literature. Oral Diseases.

[14] Do JH (2021). Enucleation of Recurrent Central Odontogenic Fibroma and Bone Regeneration of the Osseous Defect with Enamel Matrix Derivative and Bone Allograft: Case Report with 5-year Follow-up. Clin Adv Periodontics.

[15] Ruddocks LA, Nascimento AF, Bhattacharyya I, Islam MN, Cohen DM (2022). Central odontogenic fibroma in association with brown tumor of hyperparathyroidism in a patient with neurofibromatosis type 1. Oral Surg Oral Med Oral Pathol Oral Radiol.

[16] Ito N, Sakamoto S, Obayashi F, Kanda T. Central odontogenic fibroma with amyloid: a diagnostically challenging case. Int J Oral Maxillofac Surg. 2023. 10.1016/j.ijom.2023.01.017 36804052

